# Ultrasonic Synthesis and Biomedical Application of Mn_0.5_Zn_0.5_Er_x_Y_x_Fe_2−2x_O_4_ Nanoparticles

**DOI:** 10.3390/biom11050703

**Published:** 2021-05-08

**Authors:** Suriya Rehman, Munirah A. Almessiere, Ebtesam A. Al-Suhaimi, Mehwish Hussain, Maha Yousuf Bari, Syed Mehmood Ali, Suhailah S. Al-Jameel, Yassine Slimani, Firdos Alam Khan, Abdulhadi Baykal

**Affiliations:** 1 Department of Epidemic Diseases Research, Institute for Research & Medical Consultations (IRMC), Imam Abdulrahman Bin Faisal University, 31441 Dammam, Saudi Arabia; 2 Department of Biophysics, Institute for Research and Medical Consultations, Imam Abdulrahman Bin Faisal University, P.O. Box 1982, 31441 Dammam, Saudi Arabia; malmessiere@iau.edu.sa (M.A.A.); yaslimani@iau.edu.sa (Y.S.); 3 Biology Department, College of Science, Imam Abdulrahman Bin Faisal University, P.O. Box 1982, 31441 Dammam, Saudi Arabia; ealsuhaimi@iau.edu.sa; 4 Department of Public Health, College of Public Health, Imam Abdulrahman Bin Faisal University, 31441 Dammam, Saudi Arabia; mhmhussain@iau.edu.sa; 5 Department of English, Deanship of Preparatory Year, Imam Abdulrahman Bin Faisal University, 31441 Dammam, Saudi Arabia; myousuf@iau.edu.sa; 6 Department of Biomedical Engineering, College of Engineering, Imam Abdulrahman Bin Faisal University, P.O. Box 1982, 31441 Dammam, Saudi Arabia; symali@iau.edu.sa; 7 Department of Chemistry, College of Science, Imam Abdulrahman Bin Faisal University, 31441 Dammam, Saudi Arabia; ssaljameel@iau.edu.sa; 8 Department of Stem Cell Research, Institute for Research and Medical Consultations (IRMC), Imam Abdulrahman Bin Faisal University, P.O. Box 1982, 31441 Dammam, Saudi Arabia; fakhan@iau.edu.sa; 9 Department of Nano-Medicine Research, Institute for Research and Medical Consultations, Imam Abdulrahman Bin Faisal University, P.O. Box 1982, 31441 Dammam, Saudi Arabia; abaykal@iau.edu.sa

**Keywords:** ultrasonication, magnetic nanoparticles, biomedical, anti colon cancer activity, antifungal activity

## Abstract

In the present study, biocompatible manganese nanoparticles have been linked with zinc and iron molecules to prepare different derivatives of Mn_0.5_Zn_0.5_Er_x_Y_x_Fe_2−2x_O_4_ NPs (x = 0.02, 0.04, 0.06, 0.08, 0.10), using an ultrasonication approach. The structure, surface morphology, and chemical compositions of Mn_0.5_Zn_0.5_Er_x_Y_x_Fe_2−2x_O_4_ NPs were elucidated by X-ray diffractometer (XRD), High-resolution transmission electron microscopy (HR-TEM), scanning electron microscope (SEM), and Energy Dispersive X-Ray Analysis (EDX) techniques. The bioactivity of Mn_0.5_Zn_0.5_Er_x_Y_x_Fe_2−2x_O_4_ NPs on normal (HEK-293) and (HCT-116) colon cancer cell line was evaluated. The Mn_0.5_Zn_0.5_Er_x_Y_x_Fe_2−2x_O_4_ NPs treatment post 48 h resulted in a significant reduction in cells (via MTT assay, having an IC_50_ value between 0.88 µg/mL and 2.40 µg/mL). The specificity of Mn_0.5_Zn_0.5_Er_x_Y_x_Fe_2−2x_O_4_ NPs were studied by treating them on normal cells line (HEK-293). The results showed that Mn_0.5_Zn_0.5_Er_x_Y_x_Fe_2−2x_O_4_ NPs did not incur any effect on HEK-293, which suggests that Mn_0.5_Zn_0.5_Er_x_Y_x_Fe_2−2x_O_4_ NPs selectively targeted the colon cancerous cells. Using *Candida albicans*, antifungal activity was also studied by evaluating minimum inhibitory/fungicidal concentration (MIC/MFC) and the effect of nanomaterial on the germ tube formation, which exhibited that NPs significantly inhibited the growth and germ tube formation. The obtained results hold the potential to design nanoparticles that lead to efficient bioactivity.

## 1. Introduction

In recent years, many approaches were developed to customize and synthesize different metallic nanoparticles (MNPs), which are useful in drug design and delivery, experimental medicine, electrochemical sensors, electrical and electronic engineering, and biochemical sensor [1,2,3]. Globally, cancer has caught a notable attention due to the non-availability of effective therapy. Nanoparticles, especially metallic nanoparticles, are considered to be effective candidates for various biological applications due to their enhanced reactive surface area. 

Over the past few years, researchers are continuously trying to produce eco-friendly and efficient MNPs that contain different physicochemical properties, with sizes varying from 0.1 to 1000 nm. There are basically few approaches to synthesize MNPs, such as co-precipitation, hydrothermal, citrate assisted auto-combustion, microwave synthesis, reverse micelle, sol-gel, and ultrasonication [4,5,6,7,8]. However, among these techniques, ultrasonic approach is the more efficient method to synthesize the homogenized dispersed phase, small particles with better de-agglomeration capability. The method of ultrasonication, firstly, helps to produce high-purity materials, and secondly, from the economic view, it consumes less raw materials with an improved reaction rate [9].

Among synthesis approaches, ultrasonication is different because the chemical effects occur from acoustic cavitation. The implosive collapse of bubbles in the solution causes a prompt pressure pulse and high temperature in the solution [10,11]. As a result of this acoustic cavitation, local intense micro-mixing is attained [12]. The advantages of sonochemical synthesis can be given to the rate of the ultrasonic chemical reaction, which is so high due to the high number of collisions between reactant molecules. This approach does not need the usage of any other external reagents such as surfactants or capping reagents. Besides, sonochemical synthetic products have high purity, and the reaction environment is eco-friendly. Furthermore, just by varying ultrasound frequency and power, the size distribution of the products can be easily controlled. Bimetallic alloys and core-shell materials can be synthesized sonochemically at RT; otherwise, it would require high reaction temperatures and longer times [10,12].

MNPs are inorganic and zero-dimensional materials with a metal-based configuration. These NPs have gained increased importance because they can be easily manipulated using alternating current magnetic field (ACMF) and subsequently employed in various applications. Nanometer-sized MNPs exhibit intrinsic and unique properties, such as high saturation magnetization (Ms), biocompatibility, and less toxicity; in this regard, some breakthroughs have been conducted in various fields, such as industrial, environmental, analytical, and biomedical applications. In particular, MNPs have attracted attention for biomedical applications because these particles feature easy controllability, biological compatibility, physicochemical properties, and superior magnetic properties [13].

The present study synthesized different derivatives of manganese zinc nanocomposites (Mn_0.5_Zn_0.5_Dy_x_Eu_x_Fe_2−2x_O_4_) NPs, with x = 0.02, 0.04, 0.06, 0.08, 0.10, using an ultrasonication method. Subsequently, their biological activities were also evaluated by examining their anticancer and antifungal impact.

## 2. Materials and Methods

### 2.1. Synthesis of Nanoparticles

Mn_0.5_Zn_0.5_Er_x_Y_x_Fe_2−2x_O_4_ NPs (x = 0.02, 0.04, 0.06, 0.08, 0.10) were formed by ultrasonic irradiation procedure. All chemicals were obtained from Merck (Darmstadt, Germany) with high purity and used as received. The following metals were used as initial materials: nitrate and chloride, zinc nitrate (Zn(NO_3_)_2_), manganese(II) chloride (MnCl_2_·4H_2_O), iron (III) nitrate nonahydrate ((Fe(NO_3_)_3_·9H_2_O), erbium (III) nitrate hydrate (Er(NO_3_)_3_), and yttrium(III) nitrate hexahydrate (Y(NO_3_)_3_). Spinel nanoferrites of the chemical composition Mn_0.5_Zn_0.5_Er_x_Y_x_Fe_2−2x_O_4_ NPs (x = 0.02, 0.04, 0.06, 0.08, 0.10) were prepared by the sonochemical reaction technique. First, the specific amounts of metal nitrates and chlorides (batch composition of constituents) were thoroughly mixed in deionized (DI) water of 50 mL volume. Later, the different solutions were mixed with each other, and NaOH solution (2 M) was added to achieve a resultant pH of 11. Finally, the solution was exposed to ultrasonic waves (Ultrasonic Homogenizer UZ SONOPULS HD 2070) of 20 kHz frequency (power of 70 W), for 1 h. At the end of the ultrasonication process, the solution temperature was as high as 90 °C because of several collisions. Upon cooling, a fine powder was obtained, which was further washed using DI water. Finally, the blackish powder was isolated from the DI water via an external magnet and dried up at 90 °C, for 8 h, without any calcination process [14]. The structure for each composition was investigated by Rigaku Benchtop Miniflex XRD with CuKα radiation (Rgaku, Tokyo, Japan). The morphological study was imaged through SEM (FEI Titan ST) having EDX system and TEM (FEI Morgagni 268).

### 2.2. Anti-Colon Cancer Activity

#### 2.2.1. In Vitro Cytotoxicity

To study the impact of ultrasonicated Mn_0.5_Zn_0.5_Er_x_Y_x_Fe_2−2x_O_4_ NPs, we have used normal, non-cancerous cells (human embryonic kidney cells (HEK-293) and colon cancer cells (HCT-116, human colorectal carcinoma cells). Both HCT-116 and HEK-293 cells were obtained from ATCC-American Type Culture Collection, Manassas, Virginia, United States. The cell viability was measured by MTT assay, as described previously [15,16]. In brief, 70–80% confluence cells were grown with varying concentrations (2.0 µg/mL–40 µg/mL) of ultrasonicated Mn_0.5_Zn_0.5_Er_x_Y_x_Fe_2−2x_O_4_ NPs. In the control group, NPs were excluded, and, after 48 hours, the cells were incubated in MTT (Sigma-Aldrich, St. Louis, MO, USA) for 4 hours. The cells were washed and read using a microplate reader (Bio-Rad Laboratories, Hercules, CA, USA) at 570 nm. ANOVA was used to analyze the data. All of the analysis was run on GraphPad Prism software [Version 6.0]. P value less than 0.05 was taken as a significant difference in results.

#### 2.2.2. DAPI Staining

To visualize the nuclear morphology of cancer cells after NPs treatment, the cells were stained with DAPI (4′,6-diamidino-2-phenylindole), which is a fluorescent stain that binds strongly to adenine–thymine-rich regions in DNA. It is used extensively in fluorescence microscopy. Both the control and the treated cancerous cells were studied after 48 h. Post 48 h of treatment, cells were added with (4%) paraformaldehyde and later washed with (0.1%) Triton X-100, for 5 min. Then cells were stained with DAPI (1 μg/mL), for 5 min, in the dark environment, as per the previously described method [16]. The morphology of nucleus was visualized using a Confocal Scanning Microscope (Zeiss, Frankfurt, Germany) having a digital camera. 

### 2.3. Antifungal Activity 

*C. albicans* ATCC 14053 was selected for the antifungal studies. The MIC was obtained in SDB (Sabouraud’s broth), using synthesized ultrasonicated Mn_0.5_Zn_0.5_Er_x_Y_x_Fe_2−2x_O_4_ NPs ranging from 16 to 0.5 mg/mL of concentration. The initial fungal inoculum of 2.5 × 10^6^ CFU mL^−1^ was prepared by using 24 h old culture, grown at 28 °C. The prepared *Candida* with varying concentrations of NPs were further incubated at 28 °C with aeration for 24 h. The MIC is taken as the least amount of a test drug that apparently inhibits 99% growth of an organism [17]. 

#### 2.3.1. Minimal Fungicidal Concentration (MFC)

After the MIC determination of NPs, an aliquot from MIC tubes, which had no visible growth, was inoculated on fresh SDA plates (Sabouraud’s agar) and incubated for 48 h at 28 °C. The MFC is taken as the least amount of a drug that can completely kill the fungus/yeast and has a CFU of less than 3 per plate.

#### 2.3.2. Effect of Ultrasonicated NPs on Germ Tube Formation

The impact of ultrasonicated Mn_0.5_Zn_0.5_Er_x_Y_x_Fe_2−2x_O_4_ NPs on the formation of germ tube in *C. albicans* using liquid medium was studied, as per the method described by [18]. Precisely, the inoculum of C. *albicans* was prepared and adjusted, as mentioned in the previous section. Further, the *Candida* was grown in sterile RPMI 1640 broth (supplemented with sterile pooled sheep serum) added with the desired concentration of ultrasonicated Mn_0.5_Zn_0.5_Er_x_Y_x_Fe_2−2x_O_4_ NPs obtained as the MIC in the earlier experiment (16, 8, 8, 8, 4 mg/mL, for x = 0.02, 0.04, 0.05, 0.06 and 0.1, respectively). The prepared broth of *Candida* and NPs were incubated at 37 °C for 4h with aeration. Untreated *C. albicans* were taken as a negative control. Subsequently, the incubated *Candida* was used for smear preparation for microscopic visualization, and photo was captured using a light microscope (Nikon ECLIPSE Ni, New York, United States), at a magnification of 40×. The observation and presence of germ tube were manually carried out and obtained in percentage, equivalent to the total number of cells per image [18].

## 3. Results and Discussion

### 3.1. Morphological Analyses

**Figure 1:**
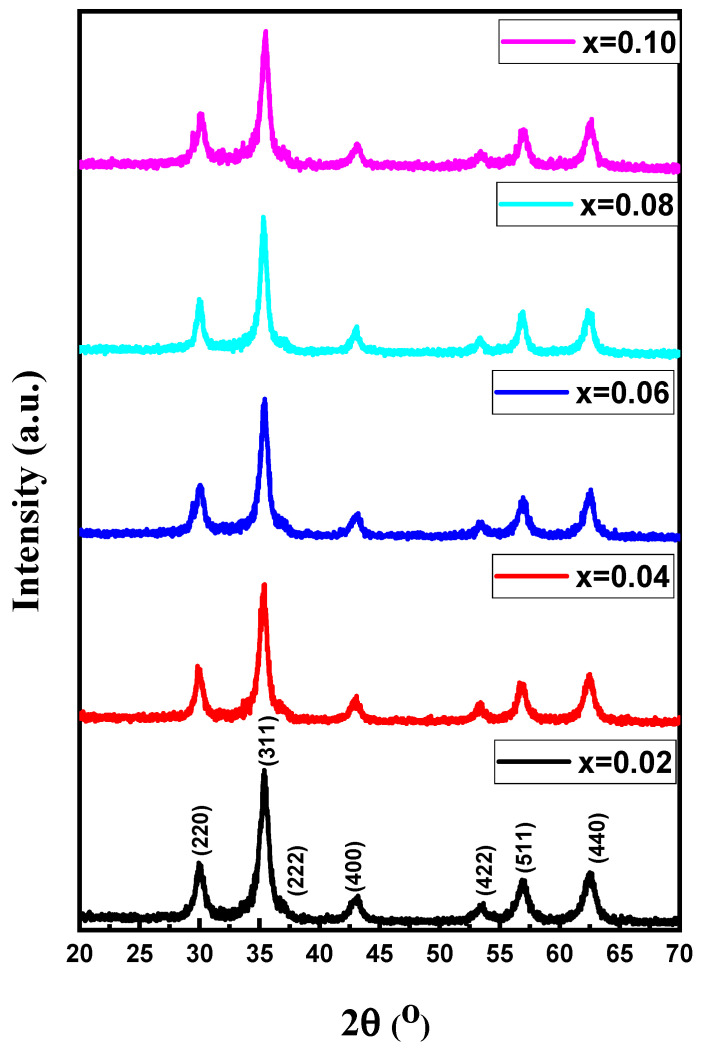
X-ray powder patterns of Mn_0.5_Zn_0.5_Er_x_Y_x_Fe_2−2x_O_4_ NPs (x = 0.02, 0.04, 0.06, 0.08, 0.10).

Figure 1 presents the X-ray powder patterns of Mn_0.5_Zn_0.5_Er_x_Y_x_Fe_2−2x_O_4_ (x = 0.02, 0.04, 0.06, 0.08, 0.1) NPs. All patterns revealed the indexed peaks of spinel ferrite. The lattice constants were estimated and found to increase with the increase in the content of substituted ions, from 8.4221 to 8.4623. The average of the crystallites’ size was estimated to be about 30 nm.

**Figure 2:**
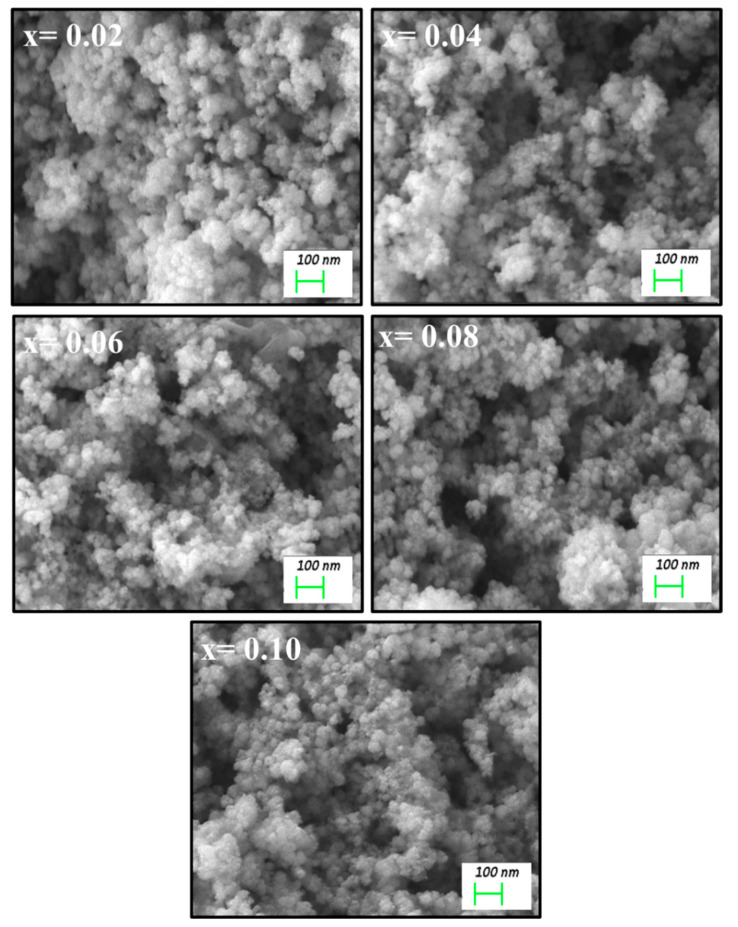

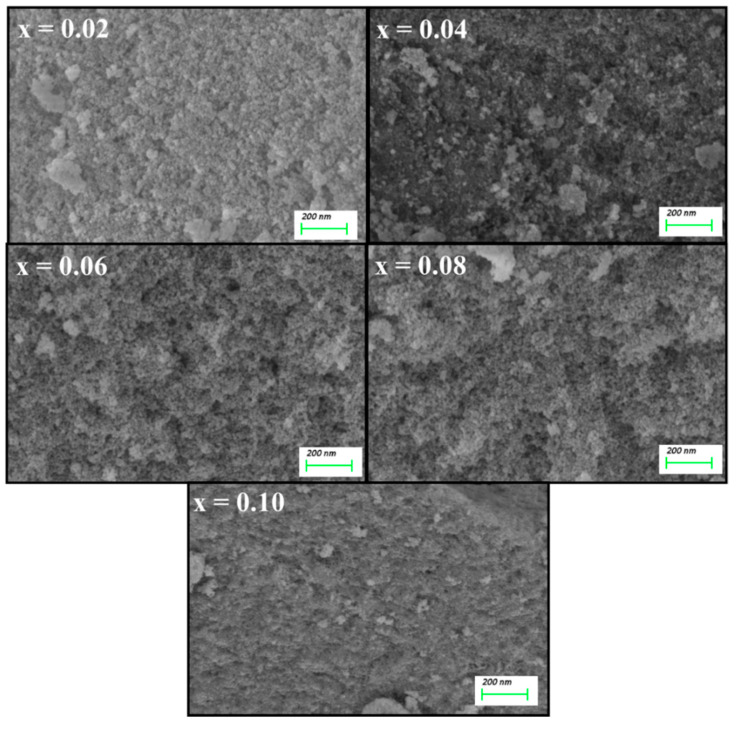
FE-SEM images of Mn_0.5_Zn_0.5_Er_x_Y_x_Fe_2−2x_O_4_ NPs (x = 0.02, 0.04, 0.06, 0.08, 0.10).

**Figure 3:**
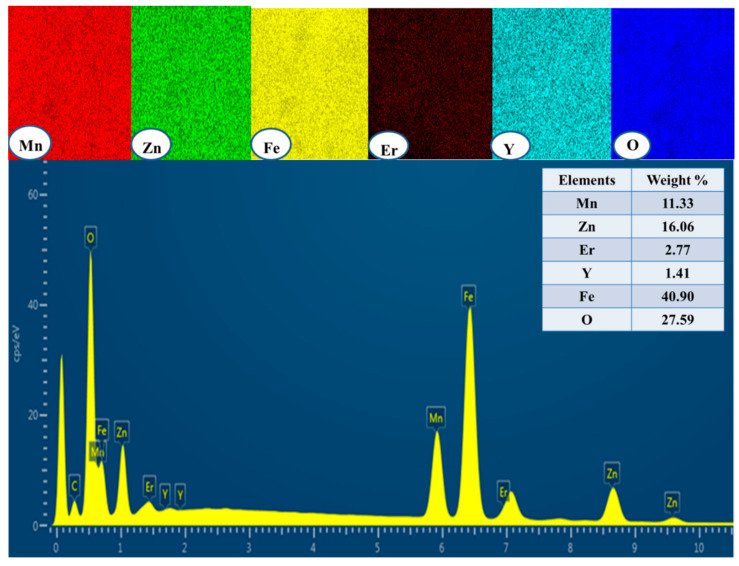
EDX and elemental mapping of Mn_0.5_Zn_0.5_Er_x_Y_x_Fe_2−2x_O_4_ NPs (x = 0.04).

**Figure 4:**
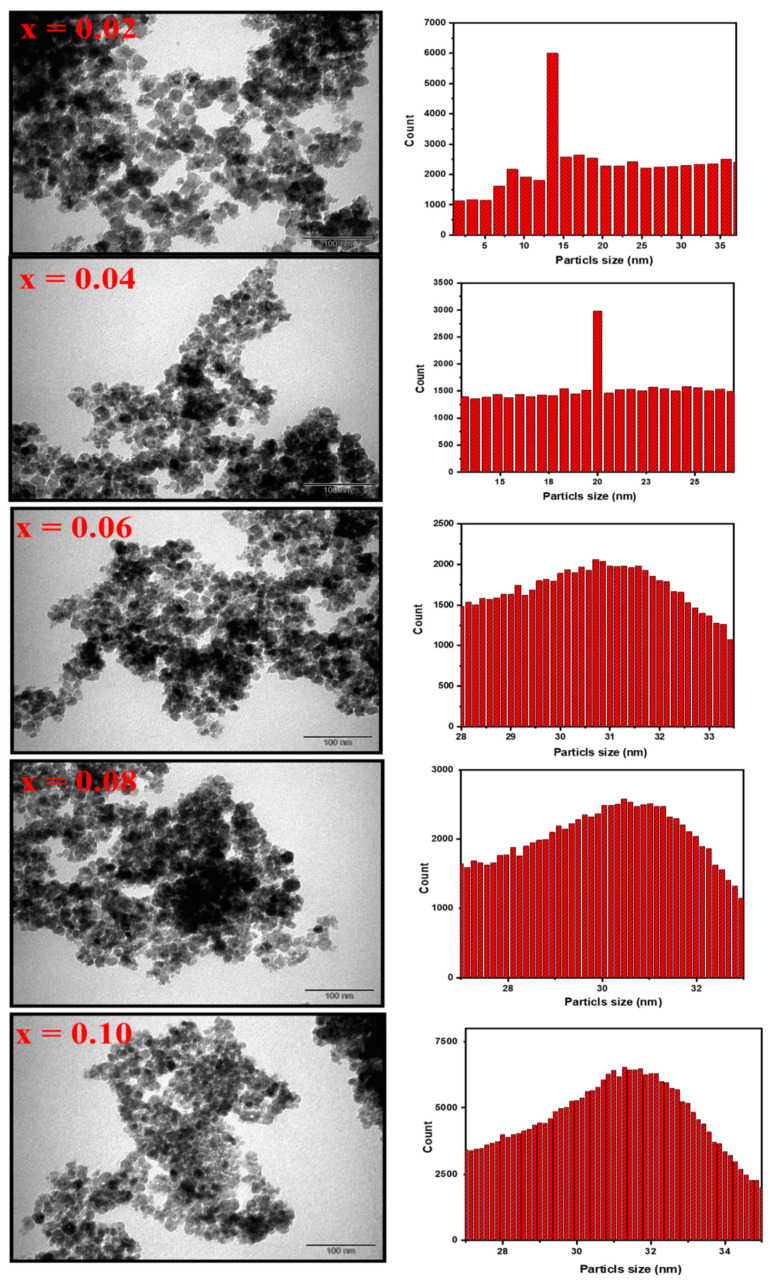
TEM images of Mn_0.5_Zn_0.5_Er_x_Y_x_Fe_2−2x_O_4_ NPs (x = 0.02, 0.04, 0.06, 0.08, 0.10) and histogram of particle size distribution.

Figure 2 shows the FE-SEM micrographs of Mn_0.5_Zn_0.5_Er_x_Y_x_Fe_2−2x_O_4_ NPs (x = 0.02, 0.04, 0.06, 0.08, 0.10). The samples revealed the assembling of cubic grains. EDX and elemental mapping offered the stoichiometric of the consisting elements of Mn_0.5_Zn_0.5_Er_x_Y_x_Fe_2−2x_O_4_ NPs (x = 0.04) NPs with no occurrence of any contamination, as observed in Figure 3. The TEM micrographs demonstrated the cubic shape and size of nanoparticles of Mn_0.5_Zn_0.5_Er_x_Y_x_Fe_2−2x_O_4_ NPs (x = 0.02, 0.04, 0.06, 0.08, 0.10), as seen in Figure 4. 

### 3.2. Anticancer Activity

Anti-proliferative activities of ultrasonicated Mn_0.5_Zn_0.5_Er_x_Y_x_Fe_2−2x_O_4_ NPs (x = 0.02, 0.04, 0.06, 0.08, 0.10) analyzed by MTT assay were used to measure NPs on cancerous cells. The cytotoxic impact of ultrasonicated Mn_0.5_Zn_0.5_Er_x_Y_x_Fe_2−2x_O_4_ NPs (x = 0.02, 0.04, 0.06, 0.08, 0.10), post 48 h of treatment, was observed, and it was found that ultrasonicated Mn_0.5_Zn_0.5_Er_x_Y_x_Fe_2−2x_O_4_ NPs (x = 0.02, 0.04, 0.06, 0.08, 0.10) inhibited the growth of HCT-116 cells. Table 1 displays the obtained inhibitory concentration (IC_50_) of different ratios. The IC_50_ of x = 0.02, 0.04, 0.06, 0.08, 0.1 NPs were 0.88, 2.40, 0.85, 0.78, and 0.45 µg/mL, respectively. Similar reports were shown in different studies, where the treatments of different types of NPs showed an IC_50_ of about 21.6 µg/mL [19], 100-200 µg/mL [20], 40 µg/mL [21] and 48 µg/mL [22] on colon cancer cells. We also determined the effects of ultrasonicated Mn_0.5_Zn_0.5_Er_x_Y_x_Fe_2−2x_O_4_ NPs (x = 0.02, 0.04, 0.06, 0.08, 0.10) on HEK-293 to assess if they produce any cytotoxic effects on normal cells. The results showed that HEK-293 cells remained unaffected post 48 h of ultrasonicated NPs treatment. 

### 3.3. Cancer Cell Nuclear Disintegration

**Figure 5:**
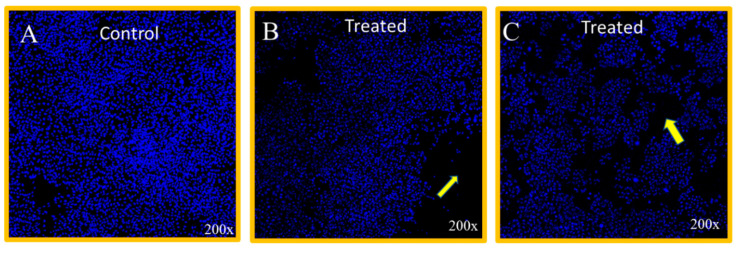
DAPI-stained cancer cell morphology. HCT-116 cells treated with ultrasonicated Mn_0.5_Zn_0.5_Er_x_Y_x_Fe_2−2x_O_4_ NPs (x = 0.02, 0.10) for 48 h. (**A**) shows the untreated cells (control); (**B)** shows NPs treatment x = 0.02 concentration 0.88 µg/mL; and (**C**) shows treatment with x = 0.10 concentration (0.45 µg/mL). Arrows in (**B**,**C**) show the nuclear disintegration. 200× magnifications.

The nuclear morphology was assessed by CLSM, which stipulated that the treatment of ultrasonicated Mn_0.5_Zn_0.5_Er_x_Y_x_Fe_2−2x_O_4_ NPs with x = 0.02 and 0.10 depicted a substantial inhibitory impact on HCT-116 cells (Figure 5B,C), compared with the control group cells (Figure 5A). 

These results suggest that ultrasonicated Mn_0.5_Zn_0.5_Er_x_Y_x_Fe_2−2x_O_4_ NPs (x = 0.02, 0.04, 0.06, 0.08, 0.10) selectively affected both breast and colon cancerous cells, and no harm was noted for the normal and healthy cells. Several reports demonstrated that magnetic nanoparticles have potential utility in drug delivery and diagnostic fields [1,23,24,25] Some studies also delineated the role of NPs in the death of cancer cells where nuclear disintegration and nuclear fragmentation were notable features [2,15,16,26,27,28]. We conclude that ultrasonically Mn_0.5_Zn_0.5_Er_x_Y_x_Fe_2−2x_O_4_ NPs hold the capability to target cancerous cells and could be considered as candidates for cancer therapy. These results are in coherence with the study of [29], who proposed the tested mixture manganese dioxide NPs as radiosensitizers. They particularly used the reaction of NPs with H_2_O_2_ (the tumor metabolite) that leads to oxygen production. The decreased hypoxia was correlated with a reduction in the radiation challenges and was therefore an enhanced antitumor influence [29]. Linking or encapsulating drug-loaded NPs to the target cells may lead to toxicity and may change the natural physiological capabilities in preserving homeostasis [30].

### 3.4. Antifungal Activities 

#### 3.4.1. MIC and MFC Determination

**Figure 6:**
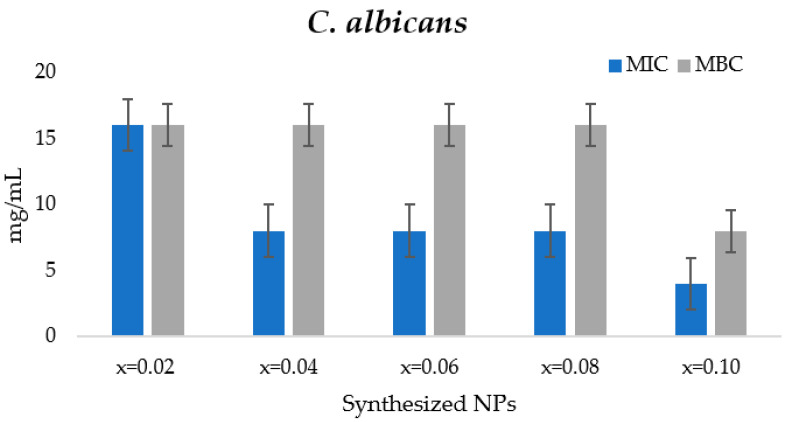
MIC and MFC of ultrasonicated Mn_0.5_Zn_0.5_Er_x_Y_x_Fe_2−2x_O_4_ NPs against *C. albicans* survival.

In the current study, the evaluation of anticandidal activity of ultrasonicated Mn_0.5_Zn_0.5_ErxYxFe_2−2x_O_4_ nanoparticles was made by determining MFC and MIC. The nanoparticles were screened at varying concentrations that ranged from 16 to 0.5 mg/mL. The obtained MIC values of ultrasonicated Mn_0.5_Zn_0.5_Er_x_Y_x_Fe_2−2x_O_4_ nanoparticles was 16, 8, 8, 8, 4 mg/mL, for x = 0.02, 0.04, 0.06, 0.08 and 0.1, respectively, whereas the MFC values were obtained as >16, 16, 16, 16, 8 mg/mL, for x = 0.02, 0.04, 0.06, 0.08 and 0.1 ultrasonicated Mn_0.5_Zn_0.5_Er_x_Y_x_Fe_2−2x_O_4_ nanoparticles, respectively (Figure 6). The obtained results depicted that antifungal activity was maximum with x = 0.1, i.e., with the increased concentration of metal substitution in the nanomaterial. Therefore, the antifungal activity of the liquid culture was concluded as the influence of the content of element Er (x = n) in the synthesized nanomaterial. Various studies have previously reported the bioactivities of metal-substituted NPs such as zinc, nickel, manganese, and copper [31,32] The influence of element Er on the antibacterial properties has been demonstrated in another report [33] against different types of strains, *Escherichia coli*, *Staphylococcus aureus*, *Enterococcus faecalis*, and *Pseudomonas aeruginosa*, with decreased levels of toxicity against the tested *Desmodesmus subspicatus*. However, the antifungal activity of this rare combination of ultrasonicated Mn_0.5_Zn_0.5_Er_x_Y_x_Fe_2−2x_O_4_ nanoparticles is the first of its kind, to the best of our knowledge. The possible explanation for this activity can be attributed to the size and shape of the synthesized ultrasonicated Mn_0.5_Zn_0.5_Er_x_Y_x_Fe_2−2x_O_4_ nanoparticles, which enable the smooth and easy contact with the candida cell surface and penetration inside the cell, leading to the cell’s death [34]. 

#### 3.4.2. Effect of Ultrasonicated Mn_0.5_Zn_0.5_Er_x_Y_x_Fe_2−2x_O_4_ NPs on Germ Tube Formation

**Figure 7:**
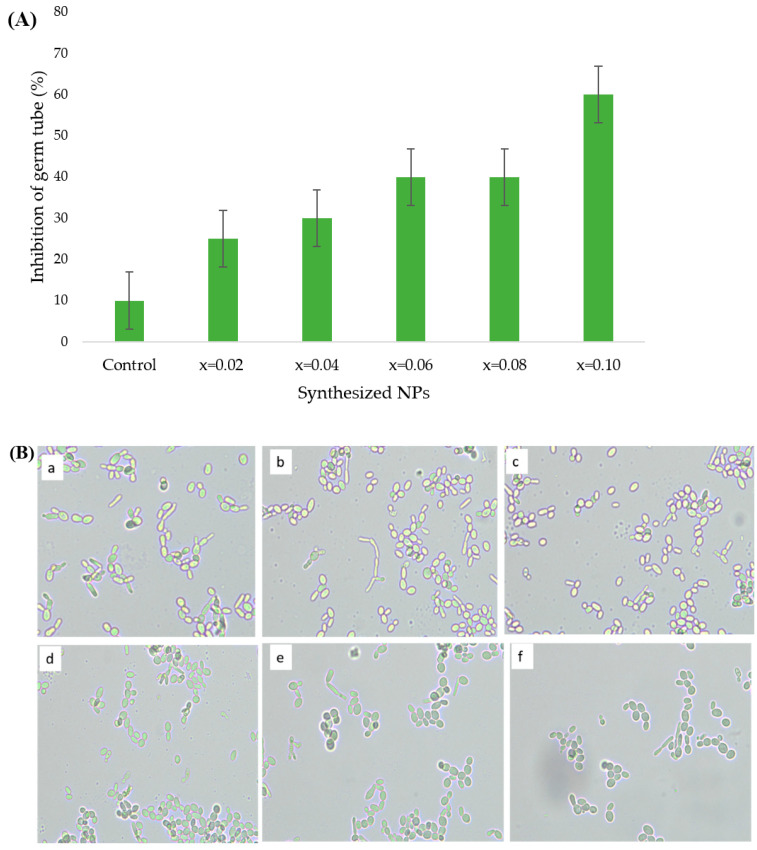
Inhibition in germ tube formation of treated *C. albicans*; (**A**) depicts the inhibition in percentage; (**B**) light microscopic images at 40× (a) control (untreated cells); (b) x = 0.02; (c) x = 0.04; (d) x = 0.06; (e) x = 0.08 and (f) x = 0.10 ultrasonicated Mn_0.5_Zn_0.5_Er_x_Y_x_Fe_2−2x_O_4_ NPs.

This assay was performed to evaluate the effect of synthesized ultrasonicated Mn_0.5_Zn_0.5_Er_x_Y_x_Fe_2−2x_O_4_ nanoparticles on germ tube formation of *C. albicans*. The results obtained during the study demonstrated that the treated *Candida* cells were inhibited in terms of growth and the formation of germ tube. The inhibition of germ tube, calculated in percentage, was found approximately 10, 25, 30, 40, 40 and 60% for untreated, x = 0.02, 0.04, 0.06, 0.08 and 0.1, ultrasonicated Mn_0.5_Zn_0.5_Er_x_Y_x_Fe_2−2x_O_4_ nanoparticles, respectively (Figure 7A). The formation of germ tube was significantly suppressed in *Candida* in the presence of x = 0.10 and moderately affected in the presence of x = 0.06 and 0.08, when compared to x = 0.02 and 0.04 untreated cells (Figure 7B). Therefore, the inhibitory action of ultrasonicated Mn_0.5_Zn_0.5_Er_x_Y_x_Fe_2−2x_O_4_ nanoparticles on the formation of germ tube of *Candida* is enhanced with the increasing ratio of element Er (x = n) in the synthesized nanomaterial. *C. albicans* is a polymorphic human pathogen that continues to form a germ tube as one of its phenotypic traits required for pathogenicity. The ability of the *Candida* to adhere to the host cell is effectively achieved by its germ tube form [35]. Furthermore, the development of germ tubes in *Candida* provides resistance against cellular responses, such as phagocytosis [36]. The inhibition of the formation of germ tube by synthesized nanomaterial could be exploited to suppress the pathogenicity in *C. albicans* for various pharmaceutical and biomedical applications.

## 4. Conclusions

This study linked manganese nanoparticles with zinc and iron molecules and prepared different derivatives of Mn_0.5_Zn_0.5_Er_x_Y_x_Fe_2−2x_O_4_ NPs using an ultrasonication approach. The surface morphology and structure of Mn_0.5_Zn_0.5_Er_x_Y_x_Fe_2−2x_O_4_ NPs were attributed by the EDX, SEM, TEM, and XRD methods. The bioactivity of Mn_0.5_Zn_0.5_Er_x_Y_x_Fe_2−2x_O_4_ NPs on normal (HEK-293) and (HCT-116) colon cancer cell line was evaluated. The Mn_0.5_Zn_0.5_Er_x_Y_x_Fe_2−2x_O_4_ NPs treatment post 48 h resulted in the significant reduction in cancer cells via MTT assay, having an IC_50_ value between 0.88 µg/mL and 2.40 µg/mL. The specificity of Mn_0.5_Zn_0.5_Er_x_Y_x_Fe_2−2x_O_4_ NPs was studied by treating them on normal cells line (HEK-293). The results showed that Mn_0.5_Zn_0.5_Er_x_Y_x_Fe_2−2x_O_4_ NPs did not incur any effect on HEK-293, which suggests that Mn_0.5_Zn_0.5_Er_x_Y_x_Fe_2−2x_O_4_ NPs selectively targeted the colon cancerous cells. Using *Candida albicans*, antifungal activity was also studied by evaluating minimum inhibitory/fungicidal concentration (MIC/MFC) and the effect of nanomaterial on the germ tube formation, which exhibited that NPs significantly inhibited the growth and germ tube formation. Based on these findings, we suggest that ultrasonicated Mn_0.5_Zn_0.5_Er_x_Y_x_Fe_2−2x_O_4_ NPs possess potential anti-cancer and anti-fungal capabilities.

## Figures and Tables

**Table 1:** Effect of Mn_0.5_Zn_0.5_Er_x_Y_x_Fe_2−2x_O_4_ NPs on cancerous cells (HCT-116) and normal cells (HEK-293).

x	IC_50_ (HCT-116	IC_50_ (HEK-293)
0.02	0.88 µg/mL	No inhibition
0.04	2.40 µg/mL	No inhibition
0.06	0.85 µg/mL	No inhibition
0.08	0.78 µg/mL	No inhibition
0.10	0.45 µg/mL	No inhibition

IC_50_ Value [µg/mL] = Inhibitory concentration (IC).
